# Prevalence of antimicrobial resistance among gram-negative isolates in an adult intensive care unit at a tertiary care center in Saudi Arabia

**DOI:** 10.4103/0256-4947.67073

**Published:** 2010

**Authors:** Sameera M. Al Johani, Javed Akhter, Hanan Balkhy, Ayman El-Saed, Mousaad Younan, Ziad Memish

**Affiliations:** From King Abdulaziz Medical City, Riyadh, Saudi Arabia

## Abstract

**BACKGROUND AND OBJECTIVES:**

Patients in the ICU have encountered an increasing emergence and spread of antibiotic-resistant pathogens. We examined patterns of antimicrobial susceptibility in gram-negative isolates to commonly used drugs in an adult ICU at a tertiary care hospital in Riyadh, Saudi Arabia.

**METHODS:**

A retrospective study was carried out of gram-negative isolates from the adult ICU of King Fahad National Guard Hospital (KFNGH) between 2004 and 2009. Organisms were identified and tested by an automated identification and susceptibility system, and the antibiotic susceptibility testing was confirmed by the disk diffusion method.

**RESULTS:**

The most frequently isolated organism was *Acinetobacter baumannii*, followed by *Pseudomonas aeruginosa, Escherichia coli, Klebsiella pnemoniae, Stenotrophomonas maltophilia,* and *Enterobacter*. Antibiotic susceptibility patterns significantly declined in many organisms, especially *A baumannii, E coli, S marcescens*, and *Enterobacter*. *A baumannii* susceptibility was significantly decreased to imipenem (55% to 10%), meropenem (33% to 10%), ciprofloxacin (22% to 10%), and amikacin (12% to 6%). *E coli* susceptibility was markedly decreased (from 75% to 50% or less) to cefuroxime, ceftazidime, cefotaxime, and cefepime. *S marcescens* susceptibility was markedly decreased to cefotaxime (100% to 32%), ceftazidime (100% to 35%), and cefepime (100% to 66%). *Enterobacter* susceptibility was markedly decreased to ceftazidime (34% to 5%), cefotaxime (34% to 6%), and pipracillin-tazobactam (51% to 35%). Respiratory samples were the most frequently indicative of multidrug-resistant pathogens (63%), followed by urinary samples (57%).

**CONCLUSION:**

Antimicrobial resistance is an emerging problem in the KFNGH ICU, justifying new more stringent antibiotic prescription guidelines. Continuous monitoring of antimicrobial susceptibility and strict adherence to infection prevention guidelines are essential to eliminate major outbreaks in the future.

Multidrug-resistant organisms (MDROs) are resistant to one or more classes of antimicrobial agents, such as β-lactams, including penicillins, cephalosporins, and monobactams, carbapenems, fluoroquinolones, and aminoglycosides. The severity and extent of disease caused by these pathogens varies by the population(s) affected and by the institution(s) in which they are found, but the prevention and control of MDROs should be a national priority.^1^ Globally, patients in the ICU have encountered an increasing emergence and spread of antibiotic-resistant pathogens. The worldwide median rate is 9 infections per 100 discharged ICU patients or 23.7 infections per 1000 patient days. Although 5% to 10% of all patients are treated in ICUs, they account for 25% of all nosocomial infections and the incidence is 5 to 10 times higher than in general hospital wards. The increased risk of infection is associated with the severity of the patient’s illness and underlying conditions, length of exposure to invasive devices and procedures, increased patient contact with healthcare personnel and length of stay in the ICU.^2^

Extended-spectrum β-lactamases (ESBLs) are often found in the *Enterobacteriaceae* family of gram-negative bacilli, particularly *Klebsiella* spp., *E coli*, and *Proteus mirabilis*; however, other gram-negative bacilli have been documented to produce such enzymes, including *Enterobacter* spp., *P aeruginosa, Citrobacter freundii, Morganella morganii,* and *S marcescens*. *P aeruginosa* has a strong tendency to become a multidrug-resistant pathogen. Usually, this process occurs as a result of both cross-infection with resistant strains and acquisition of drug resistance during treatment. *Klebsiella* spp. that produce a carbapenemase known as KPC are a serious concern because of high-level resistance against most antibiotics. The KPC enzymes are a class A, group 2f β-lactamase that efficiently hydrolyze carbapenems, as well as other β-lactam antibiotics.^3^

*A baumannii* infections have become increasingly common among critically-ill patients in intensive care units (ICUs) worldwide.^4^ *A baumannii* clinical isolates are frequently resistant to most antimicrobial agents, and evidence of pan-drug resistance among *A baumannii* isolates (i.e., resistance to all available antimicrobial agents, including polymyxins) has been reported. Data suggest that infections caused by *S maltophilia* have high mortality and that the risk factors associated with mortality are related to the initial clinical condition and patient type.^5^

These facts constitute an alarming development in the field of infectious diseases, with major public health implications. More intensive efforts are urgently required to elucidate the epidemiological and infection control issues related to these organisms and to improve the management of patients with such infections.^6^

Previous antimicrobial use is one of the strongest risk factors associated with the development of resistant pathogens. There should be monitoring of bacterial etiologies and infection patterns in order to take the necessary steps to treat infection, prevent resistance development, and lower hospital acquired cross infection. Knowledge of susceptibility patterns is helpful in selecting empirical therapy and improving the likelihood of a satisfactory outcome for the patient. The object of this study was to examine patterns of antimicrobial susceptibility of gram-negative isolates to commonly used drugs in ICU patients at a major tertiary care hospital.

## METHODS

A retrospective review was conducted of all reports of gram-negative isolates from the general adult ICU of King Fahad National Guard Hospital (KFNGH) between January 2004 and June 2009. KFNGH is an 800-bed tertiary care referral hospital in Riyadh. Gram-negative organisms were identified and tested and results were interpreted according to the guidelines of the Clinical and Laboratory Standards Institute (CLSI).^7^ Gram-negative bacilli were identified to the species level using an automated system (MicroScan Walkaway, Siemens) and confirmed using API 20E, and the antimicrobial susceptibility testing was performed using an automated system (MicroScan Walkaway, Siemens) and confirmed by the disk diffusion method or E-Test (AB Biodisk). Only one isolate per patient per year was included in the analysis.

The following antimicrobial agents were tested either by the breakpoint method (with the MicroScan system) or by the Kirby-Bauer disk diffusion method using the following disks on (Muller Hinton Agar Plate): amikacin (30-mg disks), ampicillin (10-mg disks), ceftazidime (30-mg disks), ceftriaxone (30-mg disks), ciprofloxacin (5-mg disks), gentamicin (10-mg disks), imipenem (10-mg disks), trimethoprim-sulfamethoxazole (TMP-SMX; 1.25-mg disks and 23.75-mg disks, respectively), and nitrofurantoin (300-mg disks). Quality control was performed by testing these same antimicrobials on *E coli* ATCC 25922, *E coli* ATCC 35218, *P aeruginosa* ATCC 27853, and Enterococcus faecalis ATCC 29212 to check the thymidine level on Muller Hinton Agar.^8^

The proportion of susceptible isolates was calculated as the sum of susceptible organisms (neither intermediately susceptible nor resistant) relative to the total number of organisms tested. Multidrug resistance was defined as resistance to three or more antimicrobials (imipenem, ceftazidime, ciprofloxacin, pipracillin-tazobactam, and/or an aminoglycoside). The trend in the susceptibility rate over a 6 years period (between 2004 and 2009) was calculated and analyzed to identify a statistically significant increasing or decreasing trend using chi-square for linear trend analysis. Associations between categorical variables were tested using the chi-square test. The percent of change of antibiotic susceptibility was calculated as the difference between the later (e.g. 2009) and earlier (e.g. 2004) susceptibilities percentages divided by the earlier one. All *P* values were two-tailed. *P* value <.05 was considered as significant. Open Epi software (version 2.2, Atlanta, GA, USA) was used for all statistical analyses.

## RESULTS

Of 4192 isolates taken from clinical specimens between 2004 and 2009, 2792 (66.6%) were gram-negative organisms and 1400 (33.4%) were gram-positive organisms. The number of gram-negative isolates were 310 in 2004, 481 isolates in 2005, 339 isolates in 2006, 386 isolates in 2007, 851 in 2008, and 424 in as of June 2009. The most common bacteria isolated were *Acinetobacter* spp. (886 isolates, 31.7%), followed by *P aeruginosa* (855 isolates, 30.6%), *E coli* (392 isolates, 14.0%), *K pneumonia* (285 isolates, 10.2%), *S maltophilia* (161 isolates, 5.7%), *Enterobacter* spp. (118 isolates, 4.2%), P*roteus* spp. (49 isolates, 1.8%), and 35 isolates of *Citrobacter* spp. and *Serratia marcescens* each (1.25% each). We included only samples sent to rule out infection and did not include all surveillance or screening samples, which may represent colonization rather true infection.

**Figure 1:**
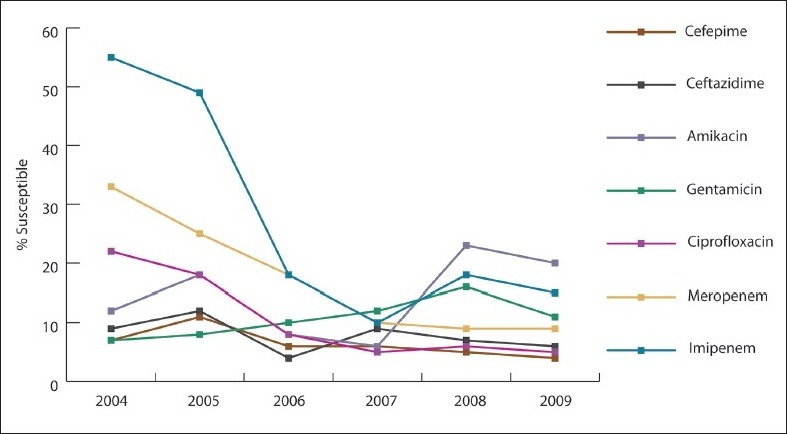
Antibiotic susceptibility for *Acintobacter baumanii* isolates tested between 2004 and 2009.

**Figure 2:**
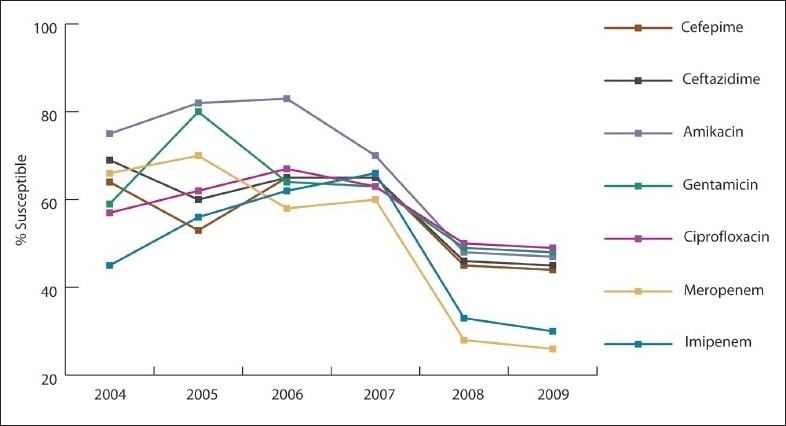
Antibiotic susceptibility pattern for *Pseudomonas aeruginosa* isolates tested between 2004 and 2009.

**Figure 3:**
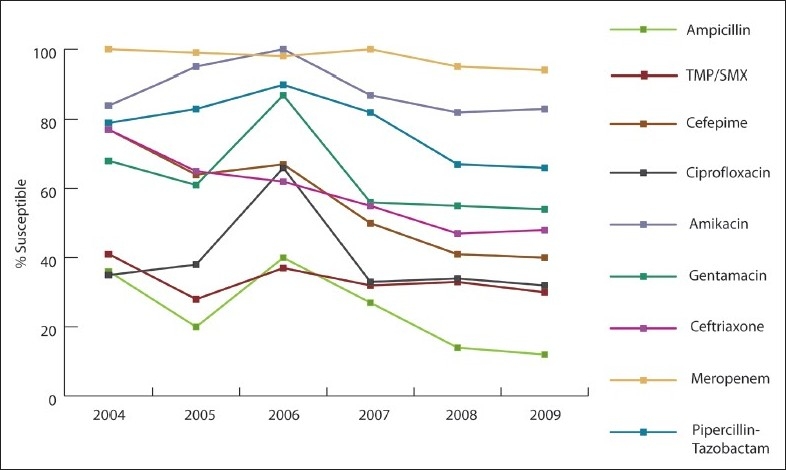
Antibiotic susceptibility pattern for *E coli* isolates tested between 2004 and 2009.

**Figure 4:**
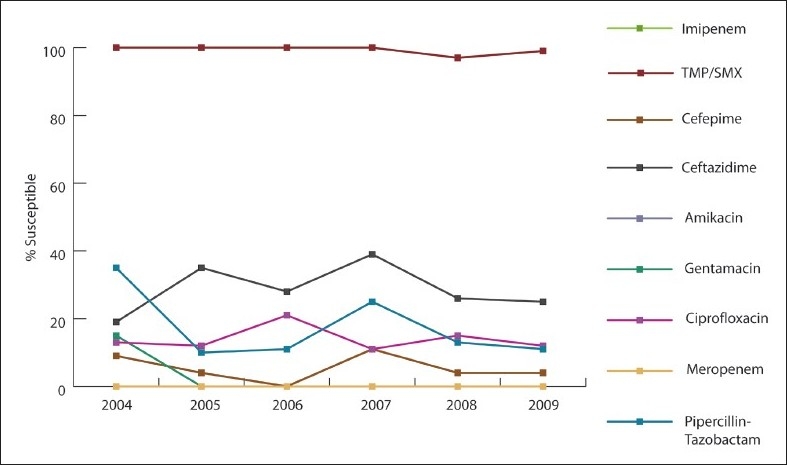
Antibiotic susceptibility for *Stenotrophomonas maltophilia* isolates tested between 2004 and 2009.

Over the study period, *A baumannii* susceptibility markedly decreased to imipenem (55% to 10%, 82% decline; *P*<.001), meropenem (33% to 10%, 70% decline; *P*<.001), ciprofloxacin (22% to 10%, 55% decline; *P*=.005), and amikacin (12% to 6%, 50% decline; *P*=.038) (Figure 1). *P aeruginosa* susceptibility significantly declined after 2007, especially for carbapenem (66% in 2004 to 26% in 2009), ceftazidime (69% in 2004 to 44% in 2009), and ciprofloxacin (67% to 49%) (Figure 2). *E coli* susceptibility markedly decreased to cefuroxime (74% to 36%, 51% decline; *P*<.001), ceftazidime (76% to 46%, 39% decline; *P*=.004), cefotaxime (76% to 46%, 39% decline; *P*=.008), cefepime (77% to 50%, 35% decline; *P*=.009), and ampicillin (36% to 27%, 25% decline; *P*=.723) (Figure 3). Of 392 *E coli* isolates, 51 isolates (16.6%) exhibited ESBL. The rate of ESBL in *E coli* increased gradually from 9% in 2004 to 16% in 2009. Of 285 *K pneumonia* isolates, the ESBL rate increased from 12% in 2004 and to 21% in 2009. The increase could have been related to better screening and detection, which was introduced to our laboratory in 2007. All of our ESBL-producing isolates were susceptible to carpabenems except one case of *K pneumonia* carpabenemase that is still under further genotypic investigation. *S marcescens* susceptibility was markedly decreased to cefotaxime (73% to 32%, 41% decline; *P*=.038), ceftazidime (91% to 35%, 61% decline; *P*=.004), and pipracillin-tazobactam (80% to 64%, 24% decline; *P*=.038). *Enterobacter* susceptibility was markedly decreased in vitro to ceftazidime (34% to 5%, 85% decline; *P*=.018), cefotaxime (34% to 6%, 82% decline; *P*=.021), and pipracillin-tazobactam (51% to 35%, 31% decline; *P*=.019). Gentamicin effectiveness in *S maltophilia* decreased dramatically, while trimethoprim-sulfamethoxazole remained the most effective antimicrobial agent (Figure 4). The most common infections were ventilator-associated pneumonia (43%) followed by urinary tract infections (32%) and blood stream infections (18%).

## DISCUSSION

Each year, healthcare-associated infections affect an estimated two million Americans, including 500 000 intensive care unit (ICU) patients, resulting in an estimated 90 000 deaths and $4.5 billion in excess health care costs.^9^ ICU patients are at increased risk of acquiring infections, most of which are associated with the use of invasive devices such as indwelling catheters, central line catheters, and mechanical ventilators.^10^

Jones et al assimilated in vitro susceptibility data from over 220000 isolates from ICUs in five countries (France, Germany, Italy, Canada, and the United States) over the period 2000 to 2002.^11^ The most frequent gram-negative species isolated from infections in the ICU was *E coli* (7.7%-15.5%), and *P aeruginosa* (10.8%-22.3%) being most common in three (USA, Canada, France) of the five countries following only *S aureus*. The gram-positive genus *Enterococcus*, either as *E faecalis*, *E faecium*, or unspeciated isolates accounted for <10% of isolates in most countries.^12^ In contrast, the most common clinical isolate in this study was *A baumannii* in addition to *E coli* and *P aeruginosa* that were also common as in other western countries. We also noted the presence of *S maltophilia* in the Saudi isolates, which is less frequent than isolates in the Western ICUs.

The resistance of some of the isolates are due to inherent resistance of the microorganisms. For example, *K pneumoniae* is naturally resistant to aminopenicillins (ampicillin). Multiresistant *A baumannii* are defined as resistant to aminoglycosides and cephalosporins: some of our isolates are also resistant to imipenem or meropenem. Rates of resistance to ceftazidime in Europe range from 15% to 97% and to imipenem from less than 1% up to 85%.^13^ * S maltophilia* is intrinsically resistant to carbapenems; however, the majority of strains are susceptible to ticarcillin-clavulanate. *S maltophilia* is frequently resistant to all aminoglycosides. Most strains of *Enterobacter* species that are resistant to third-generation cephalosporins produce an AmpC β-lactamase, which can inactivate most cephalosporins and the cephamycins. In P aeruginosa, a number of acquired β-lactamases can hydrolyze carbapenems and all other β-lactams with the exception of aztreonam. However, the frequent presence of other β-lactamases in these bacteria usually results in resistance to aztreonam.

A review of the 1999 National Nosocomial Infection Surveillance (NNIS) survey data from the CDC,^14^ involving over 300 hospitals compared with a 1994 study involving a survey of multidrug resistance among gram-negative bacilli from ICU patients in about 400 hospitals, showed that resistance to third-generation cephalosporins among *Klebsiella* isolates had increased from 7% to 9%, imipenem resistance among *P aeruginosa* strains had increased from 12% to 19%, quinolone resistance in *P aeruginosa* had increased from 12% to 23%, and resistance to third-generation cephalosporins among *Enterobacter* strains had increased from 34% to 37%. Our data showed significant resistance of cefotaxime to *Enterobacter* (66%-94%) and *E coli* (24%-54%). Pipracillin-tazobactam also exhibited increased resistance in *Citrobacter* (8%-25%) and *Enterobacter* (49%-68%).

There have been relatively few studies of antibiotic use in ICUs in Saudi Arabia. Eltahawy^15^ evaluated 100 isolates and found that *P aeruginosa*, *K pneumonia*, *E coli*, and *Enterobacter* were most commonly isolated, with imipenem, ciprofloxacin, and amikacin showing greatest efficacy. Another study comparing ICU isolates from Saudi Arabia and Kuwait^16^ showed that *E coli* and *Klebsiella* spp. demonstrated multidrug resistance to monobactams, cephems, and aminoglycosides. The Kuwaiti *Pseudomonas* spp. were more sensitive to imipenem (100% vs. 68%). They concluded that the higher number of resistant bacteria seen in Saudi Arabia might be due to greater antibiotic consumption. Another study carried out at a tertiary care hospital in Riyadh^17^ over a 1-year period showed the most frequent pathogens to be *P aeruginosa, E coli, S aureus, K pneumonia*, and *S marcescens*. In this analysis, we found that imipenem inhibited 98% of all isolates, and we had a significant overall reduction in the antibiotic susceptibility pattern in vitro.

In the 1995 European Prevalence of Infection in Intensive Care (EPIIC) Study, the most frequently reported gram-negative pathogens were *P aeruginosa, E coli*, *Acinetobacter* spp., and *Klebsiella* spp. The most commonly encountered ICU infections were ventilator-associated pneumonia, bacteremia, urinary tract infection, and wound infection. A study of clinical isolates from 19 medical centers in Canada showed that *S aureus* (MSSA and MRSA), *E coli*, *P aeruginosa, H influenzae, Enterococcus spp., S pneumoniae*, and *K pneumoniae* are the most common isolates recovered in Canadian ICUs. *Acinetobacter* spp. and *S maltophilia* are less prevalent in Europe and North America, but appear more significant in Saudi Arabia.^18^,^19^

Epidemiological information will help to implement better infection control policies in ICUs and help collaboration between different ICUs as more knowledge is gained. Therefore, developing nationwide antibiotic policy and guidelines is essential nowadays due to increasing resistance patterns. Also, developing a local antibiogram database will improve the knowledge of antimicrobial resistance patterns in Saudi Arabia and will also help to improve treatment strategies based on unit-specific data.
